# Supplementary material for: Protective population behavior change in outbreaks of emerging infectious disease
Source: BMC Infect Dis. 2021 Jun 15;21:577. doi: 10.1186/s12879-021-06299-x (PMC8205197; doi:10.1186/s12879-021-06299-x)
Supplement: Supplementary file 1 — Additional file 1: Table 1, Eq. 1: Linear Regression (DSOH ~ Epidemic Week), Stratified by Location. Table 1, Eq. 2: Linear Regression (MRR ~ Epidemic Week), Stratified by Location. Table 1, Eq. 3: Robust Linear Regression (DSOH ~ Epidemic Week), Stratified by Location. Table 1, Eq. 4: Robust Linear Regression (MRR ~ Epidemic Week), Stratified by Location. Table 1, Eq. 5: Robust Linear Regression (MRR ~ Epidemic Week + Location + Epidemic Week*Location). Table 1, Eq. 6: Robust Linear Regression (MRR ~ Serial Interval + Location + Serial Interval*Location). [file 12879_2021_6299_MOESM1_ESM.docx]

| **Table 1, Eq. 1: Linear Regression (DSOH ~ Epidemic Week), Stratified by Location** | | | | | | | |
| --- | --- | --- | --- | --- | --- | --- | --- |
|  | | | | | | | |
|  | Dependent variable: | | | | | | |
|  |  | | | | | | |
|  | Days, Symp. Onset to Hosp. | Days, Symp. Onset to Hosp. | Days, Symp. Onset to Hosp. | Days, Symp. Onset to Hosp. | Days, Symp. Onset to Hosp. | Days, Symp. Onset to Hosp. | Days, Symp. Onset to Hosp. |
|  | (1) | (2) | (3) | (4) | (5) | (6) | (7) |
|  | | | | | | | |
| Liberia Epi. Wk. | -0.31^**^ |  |  |  |  |  |  |
|  | (-0.54, -0.09) |  |  |  |  |  |  |
|  |  |  |  |  |  |  |  |
| Lofa Epi. Wk. |  | -0.31^**^ |  |  |  |  |  |
|  |  | (-0.49, -0.12) |  |  |  |  |  |
|  |  |  |  |  |  |  |  |
| Montser. Epi. Wk. |  |  | -0.31^***^ |  |  |  |  |
|  |  |  | (-0.45, -0.17) |  |  |  |  |
|  |  |  |  |  |  |  |  |
| Riyadh Epi. Wk. |  |  |  | -0.36 |  |  |  |
|  |  |  |  | (-1.53, 0.81) |  |  |  |
|  |  |  |  |  |  |  |  |
| Jeddah Epi. Wk. |  |  |  |  | -0.59 |  |  |
|  |  |  |  |  | (-1.34, 0.15) |  |  |
|  |  |  |  |  |  |  |  |
| S. Korea Epi. Wk. |  |  |  |  |  | -0.48 |  |
|  |  |  |  |  |  | (-0.96, 0.01) |  |
|  |  |  |  |  |  |  |  |
| Hong Kong Epi. Wk. |  |  |  |  |  |  | -0.35^***^ |
|  |  |  |  |  |  |  | (-0.45, -0.25) |
|  |  |  |  |  |  |  |  |
| Constant | 15.92^***^ | 7.32^***^ | 12.26^***^ | 8.14 | 6.99^*^ | 4.59^**^ | 5.93^***^ |
|  | (9.08, 22.76) | (5.42, 9.22) | (9.67, 14.86) | (-1.96, 18.24) | (2.23, 11.76) | (2.64, 6.53) | (5.08, 6.79) |
|  |  |  |  |  |  |  |  |
|  | | | | | | | |
| Observations | 43 | 18 | 38 | 11 | 10 | 6 | 16 |
| R^2^ | 0.15 | 0.41 | 0.34 | 0.04 | 0.23 | 0.48 | 0.75 |
| Adjusted R^2^ | 0.13 | 0.37 | 0.33 | -0.07 | 0.14 | 0.35 | 0.74 |
| Residual Std. Error | 52.89 (df = 41) | 7.45 (df = 16) | 22.13 (df = 36) | 12.45 (df = 9) | 5.97 (df = 8) | 3.19 (df = 4) | 4.50 (df = 14) |
| F Statistic | 7.45^**^ (df = 1; 41) | 10.89^**^ (df = 1; 16) | 18.83^***^ (df = 1; 36) | 0.36 (df = 1; 9) | 2.44 (df = 1; 8) | 3.75 (df = 1; 4) | 43.01^***^ (df = 1; 14) |
|  | | | | | | | |
| Note: | ^*^p^**^p^***^p<0.001 | | | | | | |

| **Table 1, Eq. 2: Linear Regression (MRR ~ Epidemic Week), Stratified by Location** | | | | | | | |
| --- | --- | --- | --- | --- | --- | --- | --- |
|  | | | | | | | |
|  | Dependent variable: | | | | | | |
|  |  | | | | | | |
|  | Mean Removal Rate | Mean Removal Rate | Mean Removal Rate | Mean Removal Rate | Mean Removal Rate | Mean Removal Rate | Mean Removal Rate |
|  | (1) | (2) | (3) | (4) | (5) | (6) | (7) |
|  | | | | | | | |
| Liberia Epi. Wk. | 0.01^***^ |  |  |  |  |  |  |
|  | (0.00, 0.01) |  |  |  |  |  |  |
|  |  |  |  |  |  |  |  |
| Lofa Epi. Wk. |  | 0.03^*^ |  |  |  |  |  |
|  |  | (0.01, 0.06) |  |  |  |  |  |
|  |  |  |  |  |  |  |  |
| Montser. Epi. Wk. |  |  | 0.01^***^ |  |  |  |  |
|  |  |  | (0.00, 0.01) |  |  |  |  |
|  |  |  |  |  |  |  |  |
| Riyadh Epi. Wk. |  |  |  | 0.05 |  |  |  |
|  |  |  |  | (-0.02, 0.11) |  |  |  |
|  |  |  |  |  |  |  |  |
| Jeddah Epi. Wk. |  |  |  |  | 0.03 |  |  |
|  |  |  |  |  | (-0.00, 0.07) |  |  |
|  |  |  |  |  |  |  |  |
| S. Korea Epi. Wk. |  |  |  |  |  | 0.04 |  |
|  |  |  |  |  |  | (-0.00, 0.09) |  |
|  |  |  |  |  |  |  |  |
| Hong Kong Epi. Wk. |  |  |  |  |  |  | 0.04^***^ |
|  |  |  |  |  |  |  | (0.03, 0.05) |
|  |  |  |  |  |  |  |  |
| Constant | -0.03 | -0.04 | 0.01 | -0.03 | 0.14 | 0.22 | 0.02 |
|  | (-0.08, 0.02) | (-0.31, 0.23) | (-0.07, 0.10) | (-0.62, 0.56) | (-0.09, 0.37) | (0.02, 0.41) | (-0.09, 0.12) |
|  |  |  |  |  |  |  |  |
|  | | | | | | | |
| Observations | 43 | 18 | 38 | 11 | 10 | 6 | 16 |
| R^2^ | 0.57 | 0.29 | 0.30 | 0.17 | 0.31 | 0.44 | 0.74 |
| Adjusted R^2^ | 0.56 | 0.25 | 0.28 | 0.07 | 0.22 | 0.30 | 0.72 |
| Residual Std. Error | 0.41 (df = 41) | 1.06 (df = 16) | 0.70 (df = 36) | 0.73 (df = 9) | 0.29 (df = 8) | 0.32 (df = 4) | 0.55 (df = 14) |
| F Statistic | 54.24^***^ (df = 1; 41) | 6.64^*^ (df = 1; 16) | 15.10^***^ (df = 1; 36) | 1.80 (df = 1; 9) | 3.60 (df = 1; 8) | 3.10 (df = 1; 4) | 40.44^***^ (df = 1; 14) |
|  | | | | | | | |
| Note: | ^*^p^**^p^***^p<0.001 | | | | | | |

| **Table 1, Eq. 3: Robust Linear Regression (DSOH ~ Epidemic Week), Stratified by Location** | | | | | | | |
| --- | --- | --- | --- | --- | --- | --- | --- |
|  | | | | | | | |
|  | Dependent variable: | | | | | | |
|  |  | | | | | | |
|  | Days, Symp. Onset to Hosp. | Days, Symp. Onset to Hosp. | Days, Symp. Onset to Hosp. | Days, Symp. Onset to Hosp. | Days, Symp. Onset to Hosp. | Days, Symp. Onset to Hosp. | Days, Symp. Onset to Hosp. |
|  | (1) | (2) | (3) | (4) | (5) | (6) | (7) |
|  | | | | | | | |
| Liberia Epi. Wk. | -0.20^***^ |  |  |  |  |  |  |
|  | (-0.28, -0.13) |  |  |  |  |  |  |
|  |  |  |  |  |  |  |  |
| Lofa Epi. Wk. |  | -0.32^**^ |  |  |  |  |  |
|  |  | (-0.53, -0.11) |  |  |  |  |  |
|  |  |  |  |  |  |  |  |
| Montser. Epi. Wk. |  |  | -0.21^***^ |  |  |  |  |
|  |  |  | (-0.28, -0.14) |  |  |  |  |
|  |  |  |  |  |  |  |  |
| Riyadh Epi. Wk. |  |  |  | -0.49 |  |  |  |
|  |  |  |  | (-1.26, 0.29) |  |  |  |
|  |  |  |  |  |  |  |  |
| Jeddah Epi. Wk. |  |  |  |  | -0.39^**^ |  |  |
|  |  |  |  |  | (-0.63, -0.14) |  |  |
|  |  |  |  |  |  |  |  |
| S. Korea Epi. Wk. |  |  |  |  |  | -0.48 |  |
|  |  |  |  |  |  | (-0.96, 0.01) |  |
|  |  |  |  |  |  |  |  |
| Hong Kong Epi. Wk. |  |  |  |  |  |  | -0.35^***^ |
|  |  |  |  |  |  |  | (-0.47, -0.24) |
|  |  |  |  |  |  |  |  |
| Constant | 12.29^***^ | 7.48^***^ | 10.09^***^ | 8.06^*^ | 5.42^***^ | 4.59^***^ | 6.02^***^ |
|  | (10.01, 14.57) | (5.25, 9.71) | (8.73, 11.45) | (1.37, 14.74) | (3.86, 6.98) | (2.64, 6.53) | (5.05, 6.99) |
|  |  |  |  |  |  |  |  |
|  | | | | | | | |
| Observations | 43 | 18 | 38 | 11 | 10 | 6 | 16 |
| Residual Std. Error | 12.91 (df = 41) | 8.16 (df = 16) | 8.15 (df = 36) | 6.90 (df = 9) | 0.98 (df = 8) | 3.43 (df = 4) | 3.85 (df = 14) |
|  | | | | | | | |
| Note: | ^*^p^**^p^***^p<0.001 | | | | | | |

| **Table 1, Eq. 4: Robust Linear Regression (MRR ~ Epidemic Week), Stratified by Location** | | | | | | | |
| --- | --- | --- | --- | --- | --- | --- | --- |
|  | | | | | | | |
|  | Dependent variable: | | | | | | |
|  |  | | | | | | |
|  | Mean Removal Rate | Mean Removal Rate | Mean Removal Rate | Mean Removal Rate | Mean Removal Rate | Mean Removal Rate | Mean Removal Rate |
|  | (1) | (2) | (3) | (4) | (5) | (6) | (7) |
|  | | | | | | | |
| Liberia Epi. Wk. | 0.01^***^ |  |  |  |  |  |  |
|  | (0.01, 0.01) |  |  |  |  |  |  |
|  |  |  |  |  |  |  |  |
| Lofa Epi. Wk. |  | 0.02^**^ |  |  |  |  |  |
|  |  | (0.01, 0.03) |  |  |  |  |  |
|  |  |  |  |  |  |  |  |
| Montser. Epi. Wk. |  |  | 0.01^***^ |  |  |  |  |
|  |  |  | (0.01, 0.01) |  |  |  |  |
|  |  |  |  |  |  |  |  |
| Riyadh Epi. Wk. |  |  |  | 0.02 |  |  |  |
|  |  |  |  | (-0.02, 0.06) |  |  |  |
|  |  |  |  |  |  |  |  |
| Jeddah Epi. Wk. |  |  |  |  | 0.03^**^ |  |  |
|  |  |  |  |  | (0.01, 0.06) |  |  |
|  |  |  |  |  |  |  |  |
| S. Korea Epi. Wk. |  |  |  |  |  | 0.04 |  |
|  |  |  |  |  |  | (-0.00, 0.09) |  |
|  |  |  |  |  |  |  |  |
| Hong Kong Epi. Wk. |  |  |  |  |  |  | 0.04^***^ |
|  |  |  |  |  |  |  | (0.03, 0.05) |
|  |  |  |  |  |  |  |  |
| Constant | -0.03 | 0.06 | 0.03 | 0.15 | 0.13 | 0.22^*^ | 0.02 |
|  | (-0.08, 0.02) | (-0.08, 0.19) | (-0.01, 0.08) | (-0.19, 0.48) | (-0.01, 0.28) | (0.02, 0.41) | (-0.09, 0.12) |
|  |  |  |  |  |  |  |  |
|  | | | | | | | |
| Observations | 43 | 18 | 38 | 11 | 10 | 6 | 16 |
| Residual Std. Error | 0.39 (df = 41) | 0.56 (df = 16) | 0.32 (df = 36) | 0.34 (df = 9) | 0.09 (df = 8) | 0.37 (df = 4) | 0.46 (df = 14) |
|  | | | | | | | |
| Note: | ^*^p^**^p^***^p<0.001 | | | | | | |

| **Table 1, Eq. 5: Robust Linear Regression (MRR ~ Epidemic Week + Location + Epidemic Week*Location)** | | |  |
| --- | --- | --- | --- |
|  | | |  |
|  | Dependent variable: | |  |
|  |  | |  |
|  | Mean Removal Rate | |  |
|  | | |  |
| Epidemic Week | 0.01^***^ | |  |
|  | (0.01, 0.01) | |  |
|  |  | |  |
| Lofa | 0.10 | |  |
|  | (-0.01, 0.21) | |  |
|  |  | |  |
| Montser. | 0.07 | |  |
|  | (-0.00, 0.14) | |  |
|  |  | |  |
| Riyadh | 0.18 | |  |
|  | (-0.15, 0.50) | |  |
|  |  | |  |
| Jeddah | 0.17 | |  |
|  | (-0.15, 0.49) | |  |
|  |  | |  |
| S. Korea | 0.25 | |  |
|  | (-0.00, 0.49) | |  |
|  |  | |  |
| Hong Kong | 0.04 | |  |
|  | (-0.05, 0.14) | |  |
|  |  | |  |
| Epi. Week*Lofa | 0.01^**^ | |  |
|  | (0.00, 0.02) | |  |
|  |  | |  |
| Epi. Week*Montser. | 0.00 | |  |
|  | (-0.00, 0.00) | |  |
|  |  | |  |
| Epi. Week*Riyadh | 0.01 | |  |
|  | (-0.03, 0.05) | |  |
|  |  | |  |
| Epi. Week*Jeddah | 0.03 | |  |
|  | (-0.02, 0.08) | |  |
|  |  | |  |
| Epi. Week*S. Korea | 0.04 | |  |
|  | (-0.02, 0.10) | |  |
|  |  | |  |
| Epi. Week*Hong Kong | 0.03^***^ | |  |
|  | (0.03, 0.04) | |  |
|  |  | |  |
| Constant | -0.03 | |  |
|  | (-0.08, 0.02) | |  |
|  |  | |  |
|  | | |  |
| Observations | 142 | |  |
| Residual Std. Error | 0.37 (df = 128) | |  |
|  | | |  |
| Note: | ^*^p^**^p^***^p<0.001 | |  |
|  |  | |  |
| **Table 1, Eq. 6: Robust Linear Regression (MRR ~ Serial Interval + Location + Serial Interval*Location)** | | | |
|  | | | |
|  | | Dependent variable: | |
|  | |  | |
|  | | Mean Removal Rate | |
|  | | | |
| Serial Interval | | 0.01^***^ | |
|  | | (0.01, 0.01) | |
|  | |  | |
| Lofa | | 0.10 | |
|  | | (-0.01, 0.21) | |
|  | |  | |
| Montser. | | 0.07 | |
|  | | (-0.00, 0.14) | |
|  | |  | |
| Riyadh | | 0.18 | |
|  | | (-0.15, 0.50) | |
|  | |  | |
| Jeddah | | 0.17 | |
|  | | (-0.15, 0.49) | |
|  | |  | |
| S. Korea | | 0.25 | |
|  | | (-0.00, 0.49) | |
|  | |  | |
| Hong Kong | | 0.04 | |
|  | | (-0.05, 0.14) | |
|  | |  | |
| Ser. Int.*Lofa | | 0.02^**^ | |
|  | | (0.01, 0.04) | |
|  | |  | |
| Ser. Int.*Montser. | | 0.00 | |
|  | | (-0.00, 0.01) | |
|  | |  | |
| Ser. Int.*Riyadh | | 0.01 | |
|  | | (-0.03, 0.04) | |
|  | |  | |
| Ser. Int.*Jeddah | | 0.02 | |
|  | | (-0.03, 0.07) | |
|  | |  | |
| Ser. Int.*S. Korea | | 0.03 | |
|  | | (-0.03, 0.09) | |
|  | |  | |
| Ser. Int.*Hong Kong | | 0.04^***^ | |
|  | | (0.03, 0.05) | |
|  | |  | |
| Constant | | -0.03 | |
|  | | (-0.08, 0.02) | |
|  | |  | |
|  | | | |
| Observations | | 142 | |
| Residual Std. Error | | 0.37 (df = 128) | |
|  | | | |
| Note: | | ^*^p^**^p^***^p<0.001 | |
